# Epilepsy: The future scenario

**DOI:** 10.4103/0972-2327.61269

**Published:** 2010

**Authors:** H. V. Srinivas

**Affiliations:** Sagar Hospital, 30^th^ Cross, Tilaknagar, Jayanagar, Bangalore-560 041, India. E-mail: hvsrinivas@vsnl.com

Epilepsy is a common neurological disease accounting for 1% of global burden of disease (WHO). This equals lung cancer in men and breast cancer in women.[1] In India it is estimated to have 60–80 lakhs of people with epilepsy.

Eventhough epilepsy is known to mankind for several thousands of years, it is only in the last few decades rapid advances have been made, both in diagnosis and management. What has been achieved so far and what is likely to be achieved in near future, forms the basis of my talk today.

The earlier concept based on the Institutional data was very disappointing and disheartening as the conclusion then was “epilepsy is chronic in great majority of patients requiring long periods of treatment if not for life time.” Fortunately, the subsequent neuro-epidemiological studies with community based data revealed that infact epilepsy has a better prognosis in great majority and chronic epilepsy is seen only in 15-20% and what is more important spontaneous remission is also seen in 15-20%. Talking about spontaneous remission I refer to our earlier study – the Yelandur study wherein we observed a spontaneous remission of 54%.[2] Review of the literature showed that spontaneous remission without treatment does occur upto 30% of cases.[3]

The present scenario is that 70-80% of people with epilepsy can be seizure free with antiepileptic drugs (AED)[4–6] and chronic epilepsy or medically refractory epilepsy is seen in about 20-25% of cases, which when translated into numbers will be 15-20 lakhs in India, indeed a great number. Considerable advances have been made to address this group of patients, to control the seizures. Some are already in clinical practice, some others are in the experimental stage or clinical trial stage and some more at the drawing board level.

Options for management of refractory epilepsy:

- Second line drugs
- Surgery – lesional
- Gama knife
- Surgery – non-lesional
- Seizure prediction and prevention
- Neural stimulation (Vagus, TMS, DBS)
- Gene therapy
- Stem cell therapy
- Pharmacogenetics

## a) Second Line Drugs

It is indeed disappointing that in spite of several new antiepileptic drugs entering the market at regular intervals, its effectiveness in controlling the seizures is much below expectation. The newer drugs which often times is an add on drug is said to reduce seizures by 50% in 50% of patients, the total control of seizures achieved in only 2-3%.[7] However, in a very recent study, seizure remissions (no seizures for 12 months or more) was possible in 28% and the authors concluded that the nihilistic view of intractability “if seizures are not controlled within a few years” is incorrect.[8]

## b) Surgery – Lesional

Once the newer antiepileptic drugs failed, the choice of treatment is surgical ablation of the epileptogenic area. In fact, this method of treatment is the flavor of the season. Thanks to the rapid advances in the imaging technology and the surgical skills, scenario for surgical treatment for epilepsy has changed from “last resort” to “consider it in a year after failed AED.”[7] The lesional surgery not only includes vascular malformations, but also cortical dysplasia, DNET and other tumors, more importantly, and bulks of surgical procedures are for medial temporal sclerosis. In the latter condition, the surgical results of anterior temporal lobectomy have been very gratifying, with 68% being seizure free and 24% improved.[9–11] It is likely that the surgical technique might improve further – 3D stereotactic-guided microsurgery and MRI-guided gamma knife radiosurgery with linear accelarator (LINAC) beams.[7]

## c) Gamma Knife

If someone can tackle the lesion without surgery that would definitely be most welcome, and so came in Gamma knife treatment. The greatest advantage of Gamma knife is that it is noninvasive; however there is a time gap of at least 6-10 months between the procedure and seizure control, and a need to continue anti-epileptic drugs. In one study, at 2 years’ follow up of 21 patients, 65% were seizure free.[12] The other disadvantage is the tissue is not obtained for histopathology.

## d) Surgery – Non-Lesional

While the lesional surgery has remarkably changed the scenario for intractable seizures, what if no structural lesion is observed in the imaging? Well if you cannot see a structural abnormality, try to locate functional abnormality, which is responsible for the seizures, and that is what the advancements in functional imaging technology have achieved. In unremarkable or normal MRI, a 3T MRI scanner provides useful clarification of uncertain findings in 20%[13] and in voxel-based images, yield of positive finding is 10-30%.[14] In neocortical temporal lobe epilepsy, FDG–PET shows hypometabolism in 60–70% of patients with refractory seizures and a normal MRI.[15] The ictogenic region can be identified by various methods---during ictal period MR diffusion weighted imaging, ADC, SPECT; post-ictal period DWI, ADC; interictal period FDG, PET, SISCOM and the intra operative period by newer technology of optical imaging. Also useful are functional MRI with intracranial EEG paired analysis of cerebral blood oxygen level-dependent (BOLD) signal perfusion, PET with specific ligands for *in vivo* neuro chemistry; magnetic source imaging by MEG.[16] Post-ictal diffusion MRI is more sensitive in identifying abnormal cerebral tissue than standard MRI sequences.[17] Intravenous flumazenil in the post-ictal state shows reduced ADC in hippocampi in refractive TLE.[18] SPECT is a functional imaging reflecting cerebral blood flow changes associated with the epileptogenic zone during the ictal period. FDG–PET, which images the metabolic changes in the interictal period, can localize the ictal onset zone and also optimizes selection of intracranial electrodes placement for ictal monitoring[19,20] The periictal changes are identified by SISCOM, computer-aided subtraction of periictal SPECT data from interictal SPECT data, superimposed on images of MRI brain.[21,22] Diffusion tensor imaging and tractography are used to map the white matter tracts and their relationship with epileptogenic tissue and eloquent cortex which helps surgical planning.[23] Optical imaging is one of the latest imaging techniques for intraoperative localization of epileptic foci, rolandic cortex and eloquent language regions. This identifies epileptic foci and spread of seizure activity in patients undergoing epilepsy surgery.[24,25] Thus, we see several functional imaging techniques, complementing each other to localize epileptogenic zone which can then be submitted for surgery. I am sure the technology will further fine tune to identify more number of surgically remediable lesions, when the standard structural imaging are negative or inconclusive.

## e) Seizure Prediction and Prevention

What if there is no surgically remediable lesion and the patient continues to have frequent seizures?

Seizures occurring without warning is most disabling aspect. On demand release of short acting drug or electrical stimulation during preictal state would prevent seizure.[26] Trials are being conducted for early seizure detection through implanted intracranial electrodes and prevent the seizure by responsive electrical stimulation.[27–29] Initial reports showed reduction of seizures by 50% or more in over 40% of refractory epilepsy patients.[30]

## f) Neural Stimulation (Vagus, TMS, DBS)

Vagus nerve stimulation, has been in use for quite a number of years as an add on treatment. More than 50% reduction of seizures in 50% of patients have been reported.[31]

Transcranial magnetic stimulation (TMS) is found useful in neocortical foci than the mesial temporal region. In one study of 24 patients, with 15 min twice for 1 week, 16% mean reduction was achieved in the first week, however the effect probably was short-lived.[32]

Deep brain stimulation (DBS): Success of DBS in Parkinson's disease has kindled the interest to use this method in seizure management. Stimulation has several advantages over ablation, as it is reversible or modifiable. High-frequency stimulation exerts an inhibitory effect.[33] Stereotaxic methods are used for electrode placement and connected to exterior leads in subcutaneously placed programmable stimulator, the target areas being diencephalon, cerebellum. In one study of 115 patients, 31 were seizure free, 56 improved, and no change in 27.[34] In another small study of 14 patients, improvement was observed in only two.[35] With the target on centromedian nucleus of thalamus highly significant improvement was observed in 13 patients with GTCS and atypical absence, while no benefit was seen in complex partial seizures.[36] Another report mentioned that only one out of seven patients benefited with seizure reduction.[37]

Subthalamic neural stimulation with bilateral stimulator leads in five patients reduced seizures initially by 80%; however, the subsequent follow up showed that this was maintained in two patients and there was no effect in another two patients.[38] A trial of DBS is on, consisting of Stimulation of Anterior Nucleus of the Thalamus in Epilepsy Trial (SANTE) where in electrodes are implanted in ant. nucleus of thalamus on both sides of the brain connected to single pacemaker near clavicle.[39]

## g) Gene Therapy

Parallely other methods are being explored, one of them being gene therapy. An approach to replace the defective copy of a gene with a functional copy and restore normal function in a cell population is done in haemophilia, X-linked immunodeficiency.[40] The goal of gene therapy in epilepsy is for sustained anticonvulsant effect, antiepileptogenic effect, and to block the progression of the disease. GABAergic system is the first target for gene therapy to increase GABA levels in the epileptogenic area. Implantation of genetically engineered inhibitory cells into the focus may become an option. [41] In gene therapy, delivery of genes to the brain can be through intranasal administration, stereotactic surgery. Gene delivery vehicles are by cell transplantation, cellular transduction stem cells (embryonic stem cells or adult stem cells), and viral vectors.[40]

There are more than 1000 clinical trials using gene therapy, of which 17 are for neurological diseases, e.g. Alzheimer's disease, Parkinson's disease, epilepsy. Phase I to Phase III trials are encouraging with no serious adverse effects, however not much of benefit too.

First gene therapy trail for epilepsy, xenograft of GABA expressing cells failed to show anti-epileptic effects.[40]

## h) Stem Cell Therapy

No discussion on recent advances is complete without reference to stem cell therapy. Practically, every field of medicine is looking for stem cell therapy, so much that it seems to have become one stop shop for management of every type of illness! Currently, stem cell therapy is used in oncology: Leukemia, multiple myeloma, non-Hodgkin's lymphoma, aplastic anemia, lupus, by using hematopoietic stem cells extracted from bone marrow.

Neuronal precursor cells derived from embryonic stem cells gets functionally integrated into host brain tissue after transplantation. The cells migrate into several regions of brain and become electrically active and also receive and process signals from host brain.[41] Future applications may be to replace surgically ablated neurons, to introduce cells to suppress seizures, curb epileptogenesis, and prevent chronic epilepsy after hippocampal damage related to head injury or status epilepticus[42,43]

## i) Pharmacogenetics

Let us move from clinical to basics. We are all aware that some patients simply do not respond to the AED right from the start and there are some other patients who are severely allergic to some AED, while majority can tolerate. This difference in response or sensitivity is partially due to genetic variations. The drug resistance can be due to reduced access of drugs to transfer (transporter hypothesis) or reduced drug sensitivity (target hypothesis). Multi-drugs resistance protein (P-glycoprotein) in hippocampus reduces intracellular drug concentration.[44] Hence, P-glycoprotein inhibitor with AED may be useful in drug-resistance epilepsy. The ultimate goal of pharmacogenetics is to use the genetic make up of individuals, to predict drug response and efficacy, and to predict potential adverse drug reactions. Asian patients with HLA allele HLA-B1502 are at higher risk for Stevens Johnson syndrome with Carbamazepine.[45] The day is not far off when a choice of appropriate medication can be made by use of a single simple DNA test.[46] The genetic knowledge when implemented into clinical practice facilitates diagnosis of common and rare epilepsies, optimize treatment, predicts refractoriness and drug side effects, predicts the development of epilepsy, and prevents epilepsy in appropriate cases.[47]

So far, the emphasis is on the control of seizures, i.e. antiepileptic, which is now shifted to prevention of epilepsy, i.e. antiepileptogenic which also means a cure, not just symptom control.[48] Much research is being done to understand what contributes to the development of lowered seizure threshold and also what are the compensatory mechanisms for brain to recover. Efforts are being made to identify reliable biomarkers that will lead to antiepileptogenesis therapies. At present, there are no reliable biomarkers---interictal spike wave in EEG are non-specifi, so also structural lesion on MR scan. Reliable biomarkers of epileptogenesis and epileptogenicity, when developed would be valuable for a variety of reasons to prevent the development of epilepsy, e.g. after head injury, prevent epilepsy, to identify early detection of surgically remediable epilepsy, to choose appropriate drug in a given patient.[49] Traumatic brain injury is a platform on which this can be applied to prevent the development of epilepsy, as it provides an opportunity of time interval between the injury and development of epilepsy.[50]

Until now I have been concentrating on the seizure control in the group of “refractory epilepsy.” If you look back to the initial slide you will find that in 70- 80% of people with epilepsy, the seizures are well controlled with first-line drugs. Are these people happy? The question may look ridiculous, but the fact is that the patient will answer, “yes doctor we are happy with you, for having controlled seizures, but we are not at all happy with the society we live in and their attitude towards us.” All our efforts of controlling seizures in the “refractory epilepsy” group will result in shifting them to the group of “well controlled seizures,” but the unhappiness continues! While the recent advancements have improved diagnosis and treatment, but ignorance, myths, prejudices, social stigma, and human suffering persist. A person with epilepsy is totally normal in between the seizures; hence he/she can lead a very normal life. The knowledge in the past lead to the belief that epilepsy is due to possession by devils, mental illness, hereditary, and no specific medicine available, as the result the attitude and practice was that epilepsy cannot be cured, the families are ostracised and the treatment consisted of sorcery and witchcraft. What indeed is painful and disappointing is in spite of scientific advance, and therapeutic advantages, the attitude and practice remains what it was. The societal attitude towards people with epilepsy affects education, employment, marriage, children, sports, travel and ultimately self-esteem. Law prohibiting people with epilepsy from marriage was repealed in UK in 1970, USA 1980, and in India 1999. Driving is permitted for personal use, with seizure-free period ranging from 6 to 18 months in USA, UK, Australia, and Canada. In India, driving license is not issued, even if a person is seizure free for several years. It is rightly said that “when it comes to epilepsy every country is a developing country.” International Bureau for Epilepsy (IBE) an organization for lay persons and professionals interested in the field of epilepsy came into existence in 1961. IBE addresses social problems---education, employment, marriage, insurance, driving licence restrictions, and public awareness.

Global Campaign Against Epilepsy was launched in 1997 by the joint efforts of IBE, ILAE, and WHO to bring epilepsy “Out of Shadows.” IBE has 125 members in 92 countries and India is one of the members. Indian Epilepsy Association (IEA) is formed in 1970 and presently has 27 chapters all over the country. With persistent efforts of IEA the Hindu Marriage Act was amended in 1999 so that a person with epilepsy can have a legally valid marriage. IEA now has petitioned the Government to amend the Motor Vehicle Act so as to bring the driving regulations on par with Western Countries.

Bangalore University in collaboration with CBR Network and IEA has started a Distance Education Course leading to Diploma in Epilepsy Care a one year program. This is the third country in the world to have this course. This is a value added course which benefits families with a person with epilepsy (PWE), teachers, nurses, community health care workers, teachers in special schools, and primary health care professionals including medical doctors, learning difficulty specialists, EEG technicians, and those working in NGOs, Indian epilepsy Association, Spastic Society, etc.

Ladies and gentleman we want to live in a society where everyone understands epilepsy, where attitudes are based on facts and not fiction and all of us have a social responsibility to fulfill these needs.
